# Editorial: Haptic training simulation, volume II

**DOI:** 10.3389/frobt.2022.965113

**Published:** 2022-09-08

**Authors:** Xiaojun Chen, Arnaud Lelevé, Troy McDaniel, Carlos Rossa

**Affiliations:** ^1^ Institute of Biomedical Manufacturing and Life Quality Engineering, School of Mechanical Engineering, Shanghai Jiao Tong University, Shanghai, China; ^2^ Univ Lyon, INSA Lyon, Université Claude Bernard Lyon, Ecole Centrale de Lyon, CNRS, Ampère, UMR5005, Villeurbanne, France; ^3^ The Polytechnic School, Arizona State University, Mesa, AZ, United States; ^4^ Department of Systems and Computer Engineering, Carleton University, Ottawa, ON, Canada

**Keywords:** haptic training, tactile, kinesthetic, Computer based simulation, man robot interface (MRI)

Haptic training simulation (Lelevé et al., 2020) usually deals with kinesthetic feedback. This second edition explores complementary approaches in the medical domain (as in He et al. (2022) specifically for tissue examination), to provide realistic feedback and objective assessment to trainees during their training. Thus, Dragunasu et al. and Rørvik et al. introduce novel tactile devices while Gautier et al. propose to equip practice boxes with a vision system to enable objective assessment. From an complementary point of view, Jourdes et al. propose to train on surgical robots that do not provide haptic feedback using visual feedback (Bresler et al., 2020).

Simulating effective haptic feedback in a virtual environment is a challenging problem that involves a myriad of design considerations. To improve the wearability of such devices, primary design considerations involve optimizing the footprint of the actuating mechanism on the skin and the number of actuators used to simulate haptic feedback. Using parallel actuation mechanisms to solve the problem of achieving efficient and relevant force-feedback is an actively researched topic. There have been numerous designs that seek to address this issue, but few that involve the palm of hand. Dragunasu et al. propose a novel design for a device that simulates cutaneous feedback at the palm of hand by leveraging grasping biomechanics and using tendon-like mechanical actuation to generate both tangential and normal force feedback at the palm. To show the efficacy of the haptic feedback from the present prototype, the work demonstrates a simple virtual scenario for interacting with objects.

Medical palpation training is essential to improve tactile diagnostic skills among professionals, but currently available training equipment lacks commercial viability and richness of tactile stimulation to provide realistic feedback to trainees. To address this problem, it is necessary to effectively simulate the hardness and shape of the interacting surface for palpation training. Rørvik et al. demonstrate a novel mechanism that exploits ferrogranular jamming to generate haptic feedback on a surface. Granular jamming has been previously used in soft robotic actuation mechanisms but seldom used to generate haptic feedback. This work proposes a technique to exploit this actuation mechanism by using ferromagnetic granules to provide surface haptic feedback. In addition to designing and testing the efficacy of this technique, validation studies for palpation training are also presented that show promising results. Further research in this direction might lead to impactful outcomes in the area of tactile medical training simulators.

Previous solutions are adequate for advanced computer-based simulators. Yet, large and realistic laparoscopy computer-based simulators are costly, limiting access for both universities and trainees. This necessitates the need to train (at first) on inexpensive simulation kits to acquire the basic skills. These kits are generally training boxes that enable trainees to use real instruments while working on real materials. The haptic feedback is natural, but this approach lacks immediate feedback to provide automated self-assessment facilities as it is only based on the supervision of experts. The proposal of Gautier et al. is a visual tracking algorithm using images taken inside a physical laparoscopy training box equipped with a single fish-eye camera within. The algorithm estimates in real-time the 3D positions of the laparoscopic instrument tips to feed an automated self-assessment algorithm based on tool trajectory. The interest of this approach is the reduced cost compared to usual training facilities as it only requires installing a camera inside a box, colored tape on the tools, and a computer to provide feedback and assessment without the need for continuous visual supervision by a trainer. This fills the gap between large audience basic simulators and costly computer-based ones providing rich gesture feedback and assessment. Next works will consist of using the images from more usual embedded webcams and improving the assessment algorithm.

Nevertheless, on current surgical robots such as the Da Vinci, haptic feedback is rarely available. This results in novices applying unnecessarily high forces during operations. They require considerable training on surgical robots to determine through visual feedback the relation between tissue deformation upon contact and applied forces to correctly dose their gestures. Some training out of the operating room would be less costly and could be more efficient if realistic enough. Thus, Jourdes et al. introduce a “non-haptic” suture simulator to provide such training. They worked on the wire model and the various contacts of the wire and its environment to render realistic knot tightening associated with visual cues based on implied mechanical forces or constraints to support learning how to dose the forces. These are preliminary results that still require enhancement (integrating the wire plasticity for instance) and evaluation in terms of training efficiency, but this application highlights that haptic feedback can also be transmitted through visual feedback. One can also imagine adding real kinesthetic feedback and afford trainees a way to train on suturing with more data for objective assessment and even assistance in the first training stages.

This Research Topic showcases the diversity of the approaches when dealing with haptic simulation and promising insights. The fact that nowadays only a few surgical robots provide haptic feedback motivates further research on haptic training simulators. Indeed, the next generations of these robots will provide haptic feedback and surgeons will have to train on simulated models before operating on real patients. Moreover, even if the papers of this edition are only in the medical field, one has to consider that many other domains [driving, construction machine piloting, assembly, among many other areas, see for instance (Knoke and Thoben, 2021)] still require training on gestures and dosing ones’ forces. Just think how civil aviation transformed its training approach. As soon as training simulation can avoid wounds and deaths, it should be considered as a mandatory step. It also has an economic interest as soon as training and assessing on simulators becomes less costly than on real devices. However, to adopt it widely, researchers still have some work to make them more efficient in terms of learning. This can be reached through more realistic and more immersive innovations, but not uniquely. We can observe in these papers that simple approaches could also be effective and less expensive, and so, potentially widely spread. Therefore, research directions of this topic are not only based on technological improvements of haptic and multimodal cues [visual, kinesthetic, tactile (He et al., 2022)], but also on the methods and algorithms to extract gestures and enable their objective assessment.
